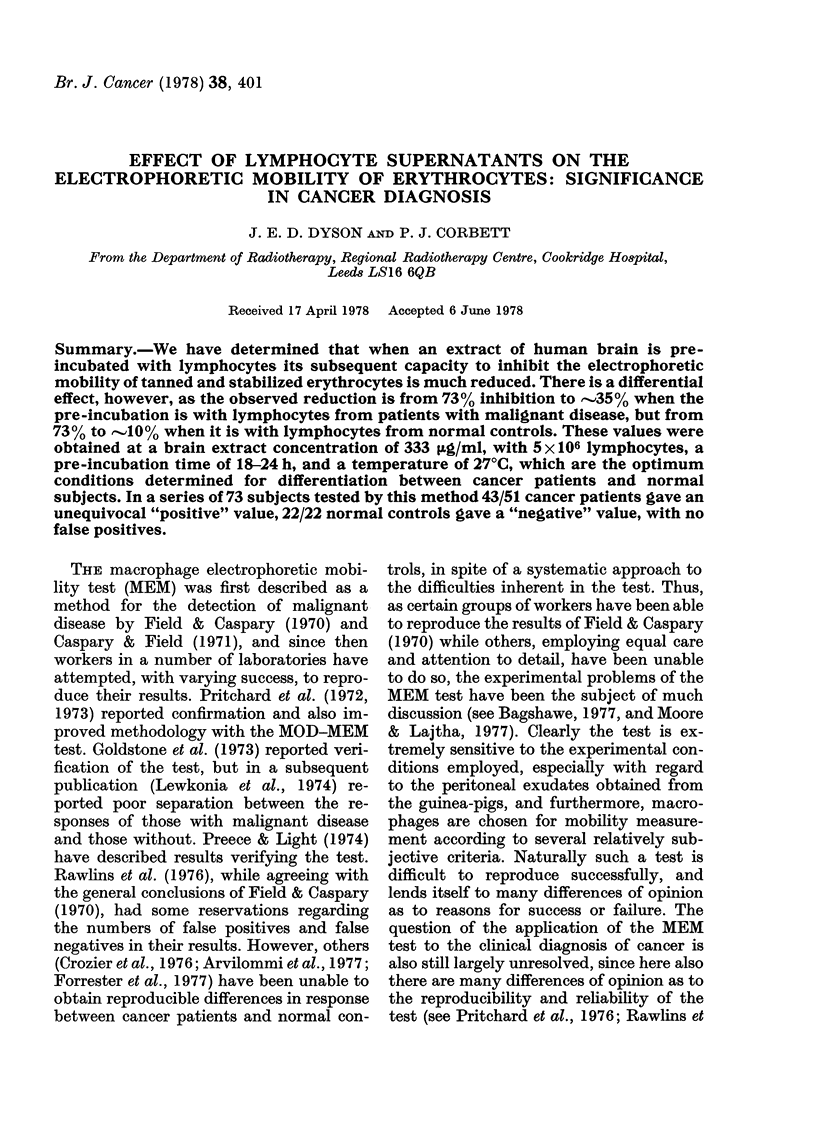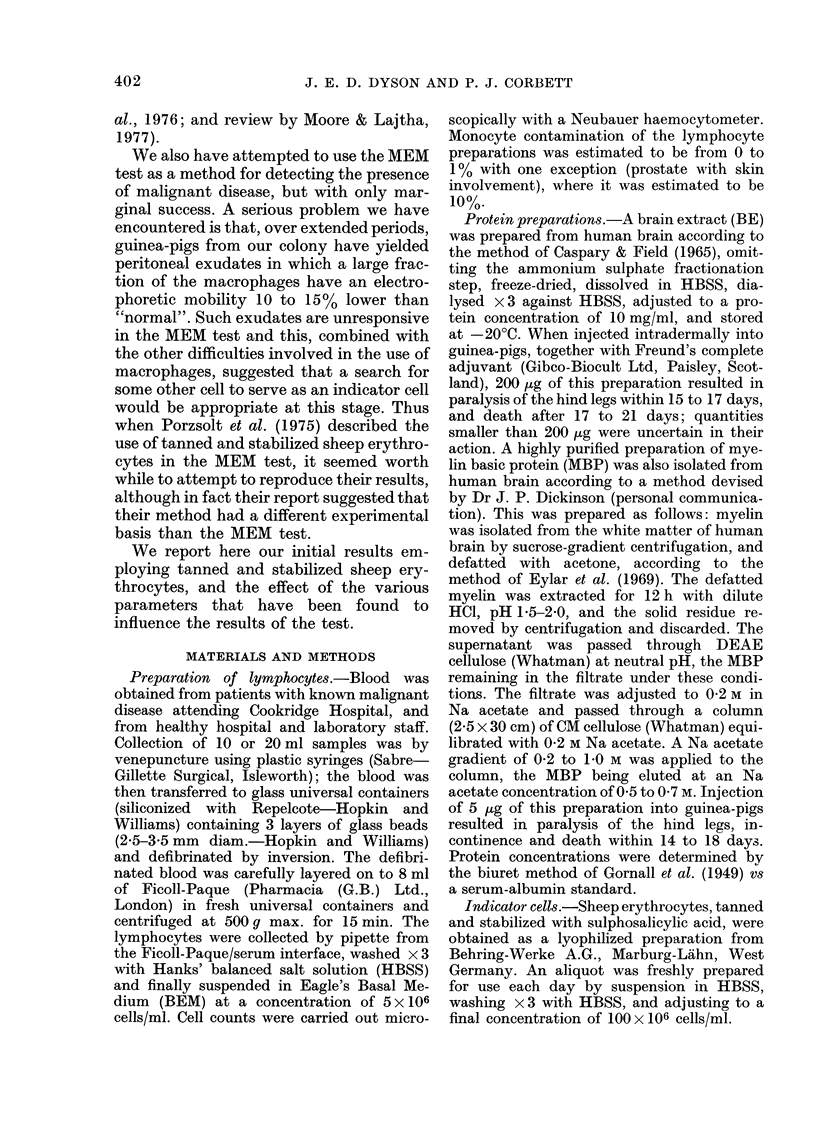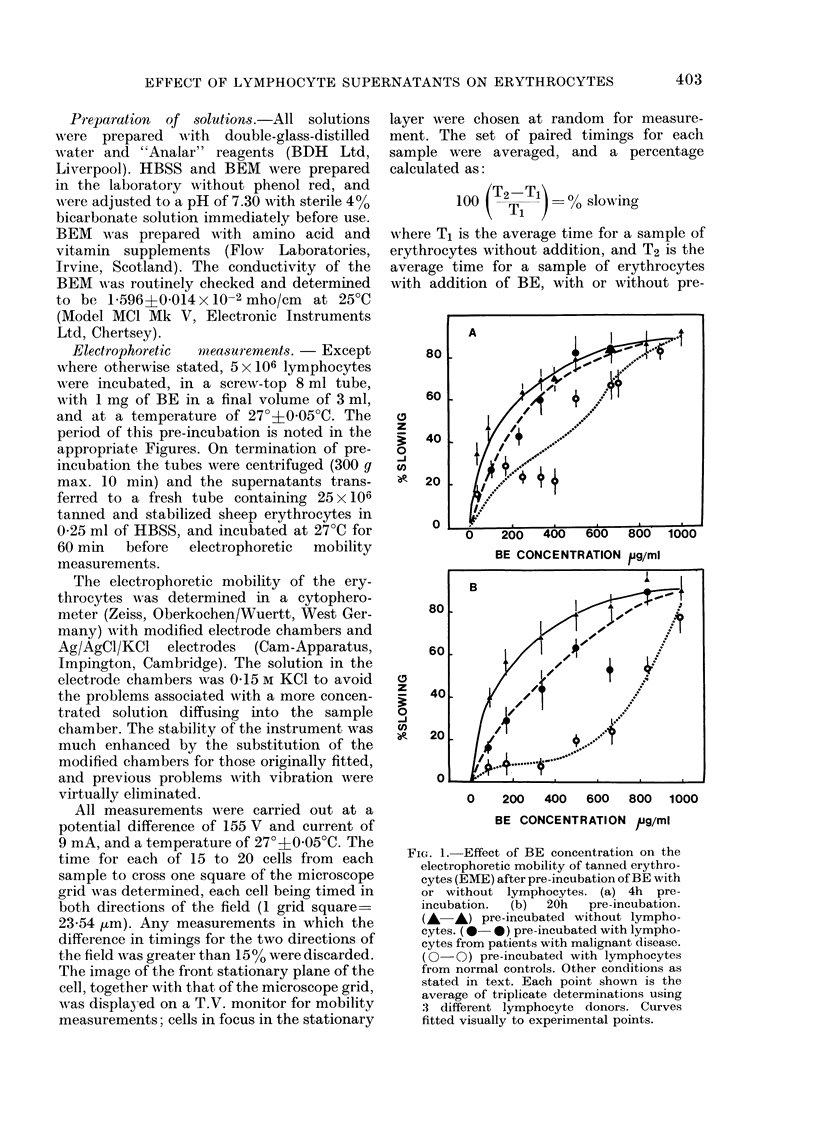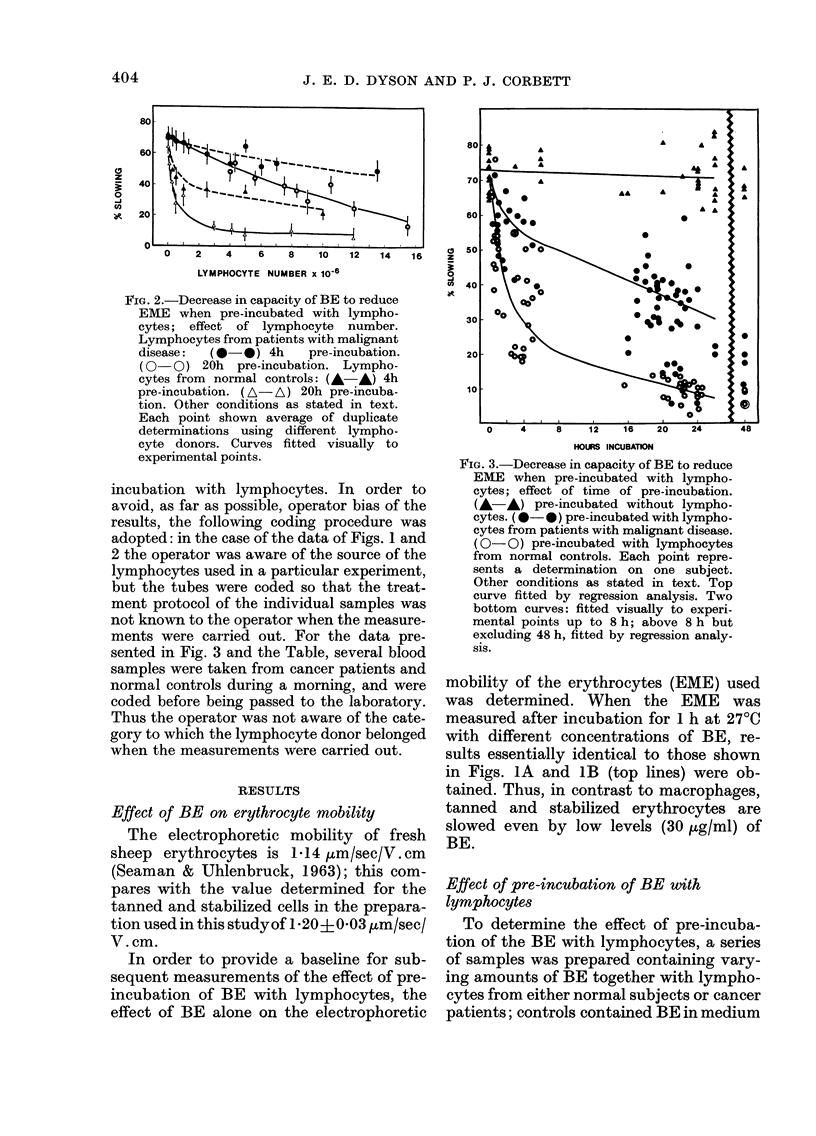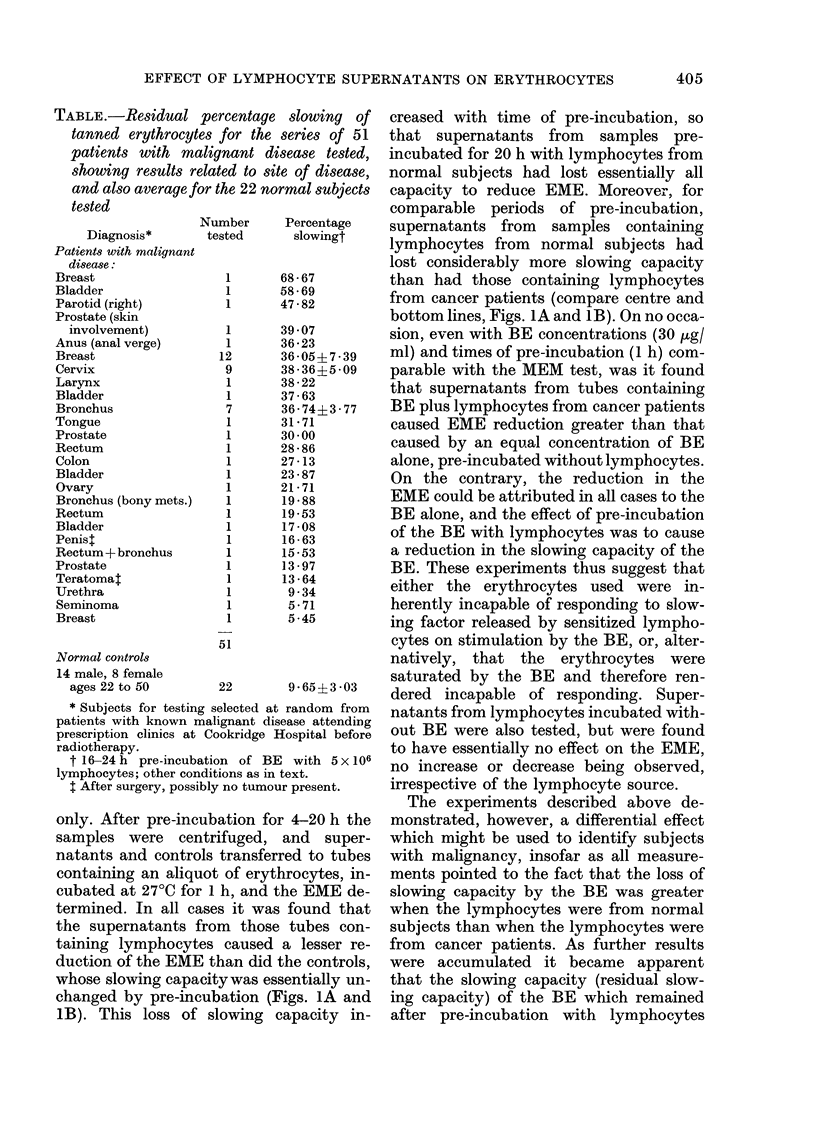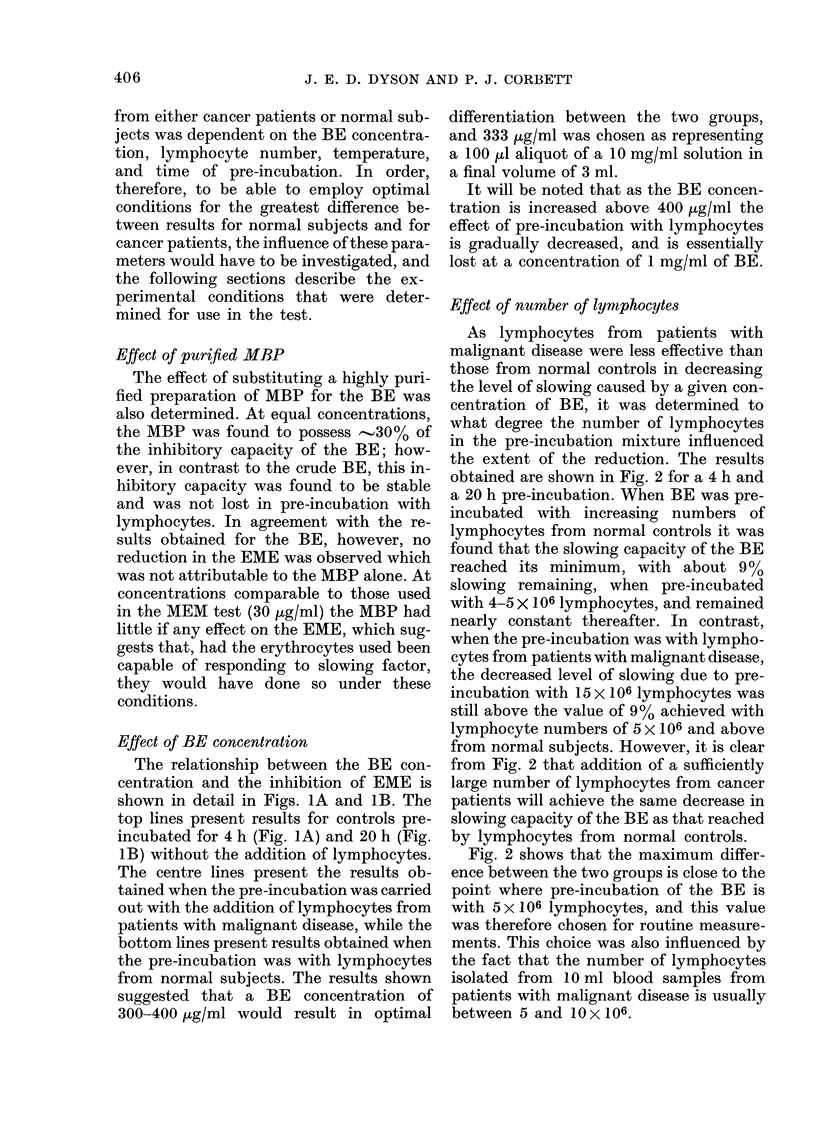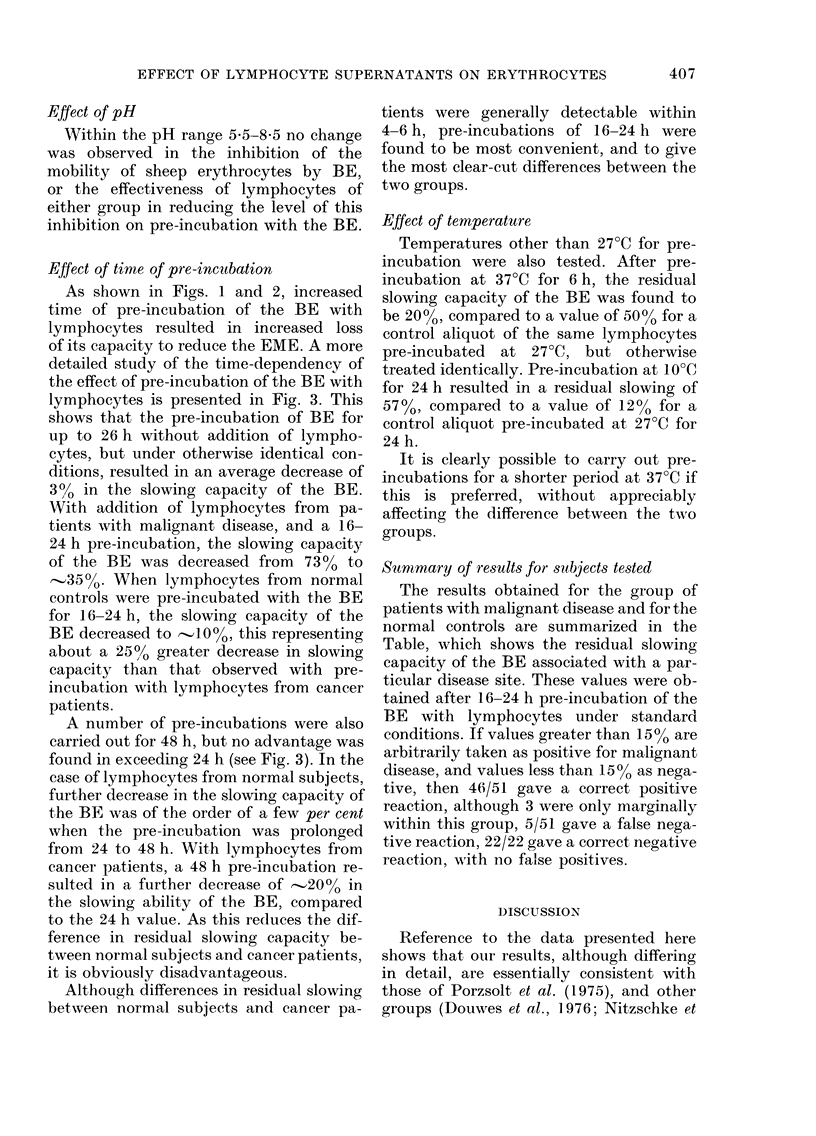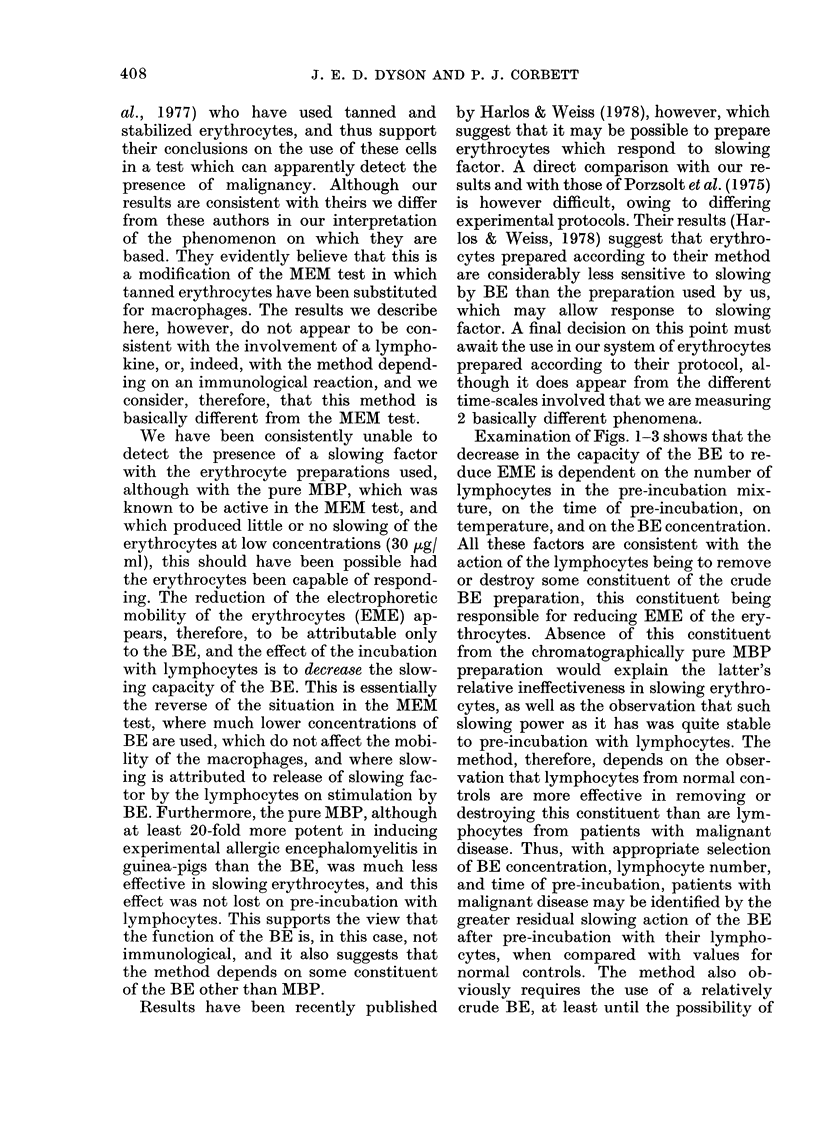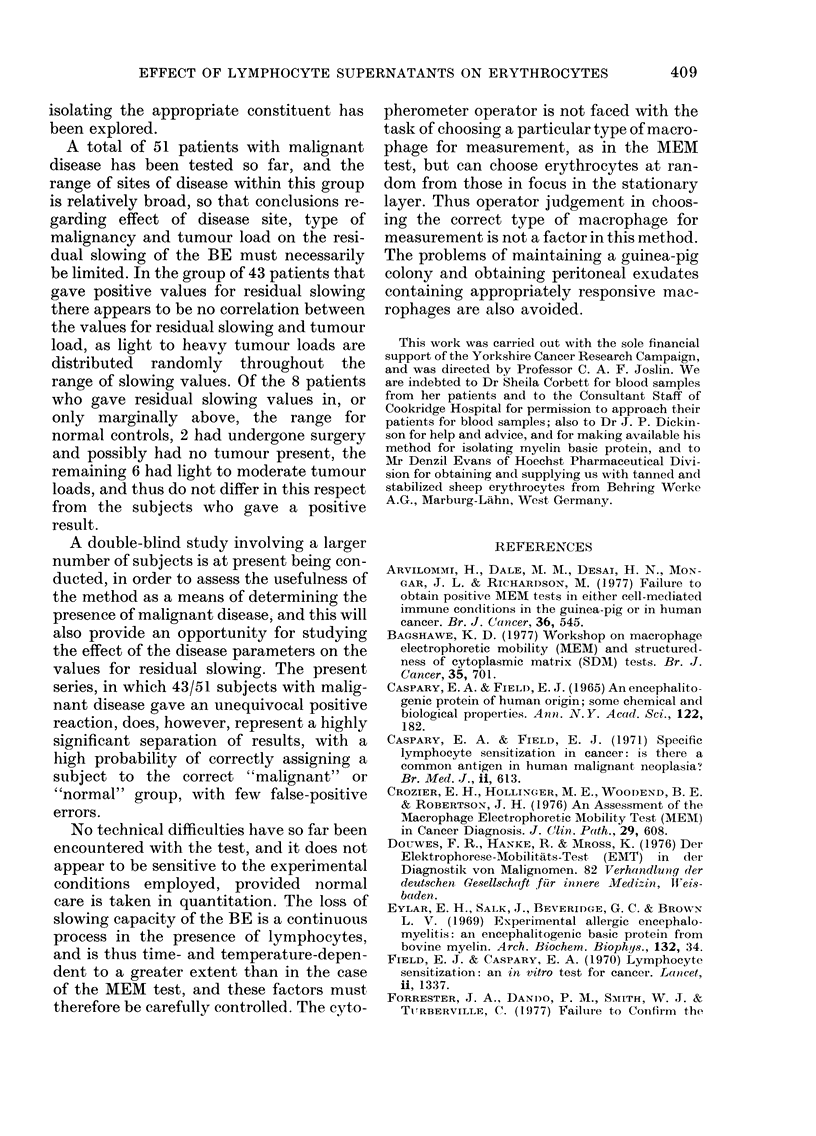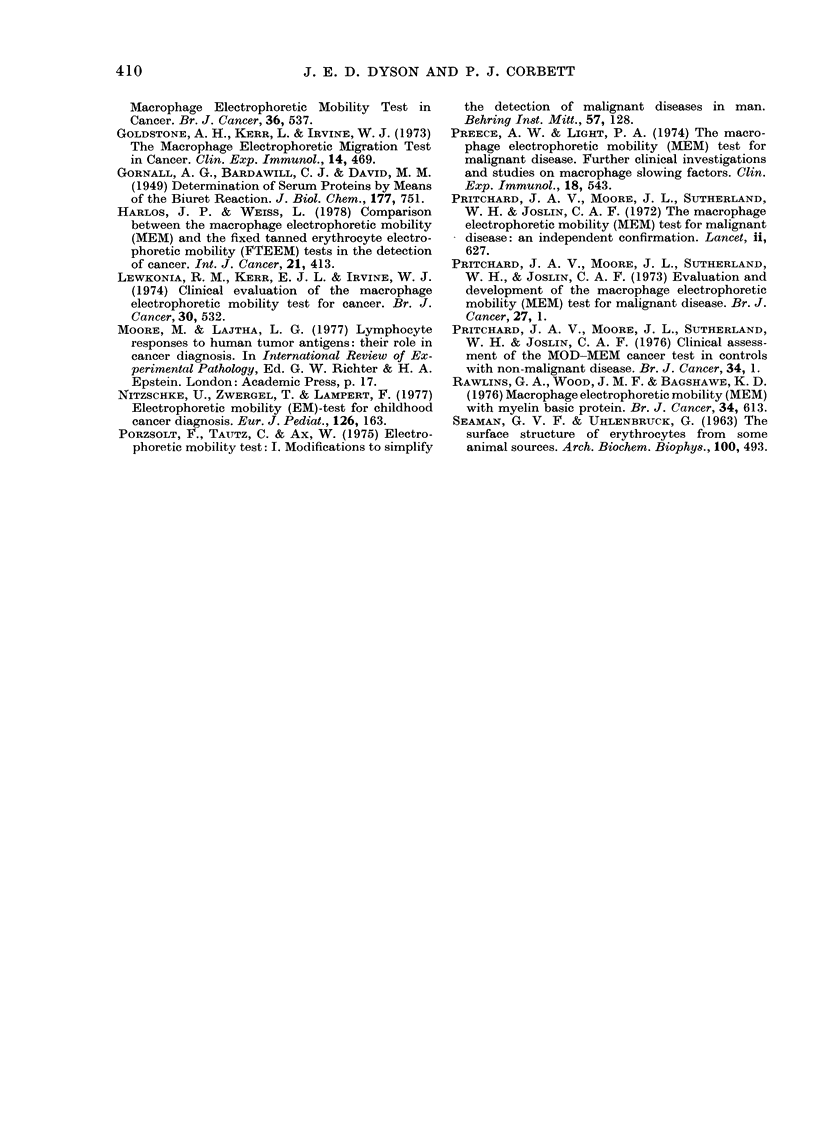# Effect of lymphocyte supernatants on the electrophoretic mobility of erythrocytes: significance in cancer diagnosis.

**DOI:** 10.1038/bjc.1978.221

**Published:** 1978-09

**Authors:** J. E. Dyson, P. J. Corbett

## Abstract

We have determined that when an extract of human brain is preincubated with lymphocytes its subsequent capacity to inhibit the electrophoretic mobility of tanned and stabilized erythrocytes is much reduced. There is a differential effect, however, as the observed reduction is from 73% inhibition to approximately 35% when the pre-incubation is with lymphocytes from patients with malignant disease, but from 73% to approximately 10% when it is with lymphocytes from normal controls. These values were obtained at a brain extract concentration of 333 microgram/ml, with 5 times 10(6) lymphocytes, a pre-incubation time of 18-24 h, and a temperature of 27 degrees C, which are the optimum conditions determined for differentiation between cancer patients and normal subjects. In a series of 73 subjects tested by this method 43/51 cancer patients gave an unequivocal "positive" value, 22/22 normal controls gave a "negative" value, with no false positives.


*Br. J. Cancer* (1978) **38**, 401

### EFFECT OF LYMPHOCYTE SUPERNATANTS ON THE ELECTROPHORETIC MOBILITY OF ERYTHROCYTES: SIGNIFICANCE IN CANCER DIAGNOSIS

J. E. D. DYSON AND P. J. CORBETT

*From the Department of Radiotherapy, Regional Radiotherapy Centre, Cookridge Hospital, Leeds LS16 6QB*

Received 17 April 1978 Accepted 6 June 1978

**Summary.—We have determined that when an extract of human brain is pre-incubated with lymphocytes its subsequent capacity to inhibit the electrophoretic mobility of tanned and stabilized erythrocytes is much reduced. There is a differential effect, however, as the observed reduction is from 73% inhibition to ~35% when the pre-incubation is with lymphocytes from patients with malignant disease, but from 73% to ~10% when it is with lymphocytes from normal controls. These values were obtained at a brain extract concentration of 333 μg/ml, with $5 \times 10^6$ lymphocytes, a pre-incubation time of 18–24 h, and a temperature of 27°C, which are the optimum conditions determined for differentiation between cancer patients and normal subjects. In a series of 73 subjects tested by this method 43/51 cancer patients gave an unequivocal "positive" value, 22/22 normal controls gave a "negative" value, with no false positives.**

THE macrophage electrophoretic mobility test (MEM) was first described as a method for the detection of malignant disease by Field & Caspary (1970) and Caspary & Field (1971), and since then workers in a number of laboratories have attempted, with varying success, to reproduce their results. Pritchard *et al.* (1972, 1973) reported confirmation and also improved methodology with the MOD–MEM test. Goldstone *et al.* (1973) reported verification of the test, but in a subsequent publication (Lewkonia *et al.*, 1974) reported poor separation between the responses of those with malignant disease and those without. Preece & Light (1974) have described results verifying the test. Rawlins *et al.* (1976), while agreeing with the general conclusions of Field & Caspary (1970), had some reservations regarding the numbers of false positives and false negatives in their results. However, others (Crozier *et al.*, 1976; Arvilommi *et al.*, 1977; Forrester *et al.*, 1977) have been unable to obtain reproducible differences in response between cancer patients and normal controls, in spite of a systematic approach to the difficulties inherent in the test. Thus, as certain groups of workers have been able to reproduce the results of Field & Caspary (1970) while others, employing equal care and attention to detail, have been unable to do so, the experimental problems of the MEM test have been the subject of much discussion (see Bagshawe, 1977, and Moore & Lajtha, 1977). Clearly the test is extremely sensitive to the experimental conditions employed, especially with regard to the peritoneal exudates obtained from the guinea-pigs, and furthermore, macrophages are chosen for mobility measurement according to several relatively subjective criteria. Naturally such a test is difficult to reproduce successfully, and lends itself to many differences of opinion as to reasons for success or failure. The question of the application of the MEM test to the clinical diagnosis of cancer is also still largely unresolved, since here also there are many differences of opinion as to the reproducibility and reliability of the test (see Pritchard *et al.*, 1976; Rawlins *et*

*al.*, 1976; and review by Moore & Lajtha, 1977).

We also have attempted to use the MEM test as a method for detecting the presence of malignant disease, but with only marginal success. A serious problem we have encountered is that, over extended periods, guinea-pigs from our colony have yielded peritoneal exudates in which a large fraction of the macrophages have an electrophoretic mobility 10 to 15% lower than "normal". Such exudates are unresponsive in the MEM test and this, combined with the other difficulties involved in the use of macrophages, suggested that a search for some other cell to serve as an indicator cell would be appropriate at this stage. Thus when Porzsolt *et al.* (1975) described the use of tanned and stabilized sheep erythrocytes in the MEM test, it seemed worth while to attempt to reproduce their results, although in fact their report suggested that their method had a different experimental basis than the MEM test.

We report here our initial results employing tanned and stabilized sheep erythrocytes, and the effect of the various parameters that have been found to influence the results of the test.

### MATERIALS AND METHODS

*Preparation of lymphocytes.*—Blood was obtained from patients with known malignant disease attending Cookridge Hospital, and from healthy hospital and laboratory staff. Collection of 10 or 20 ml samples was by venepuncture using plastic syringes (Sabre—Gillette Surgical, Isleworth); the blood was then transferred to glass universal containers (siliconized with Repelcote—Hopkin and Williams) containing 3 layers of glass beads (2·5–3·5 mm diam.—Hopkin and Williams) and defibrinated by inversion. The defibrinated blood was carefully layered on to 8 ml of Ficoll-Paque (Pharmacia (G.B.) Ltd., London) in fresh universal containers and centrifuged at 500 *g* max. for 15 min. The lymphocytes were collected by pipette from the Ficoll-Paque/serum interface, washed $\times 3$ with Hanks' balanced salt solution (HBSS) and finally suspended in Eagle's Basal Medium (BEM) at a concentration of $5 \times 10^6$ cells/ml. Cell counts were carried out microscopically with a Neubauer haemocytometer. Monocyte contamination of the lymphocyte preparations was estimated to be from 0 to 1% with one exception (prostate with skin involvement), where it was estimated to be 10%.

*Protein preparations.*—A brain extract (BE) was prepared from human brain according to the method of Caspary & Field (1965), omitting the ammonium sulphate fractionation step, freeze-dried, dissolved in HBSS, dialysed $\times 3$ against HBSS, adjusted to a protein concentration of 10 mg/ml, and stored at −20°C. When injected intradermally into guinea-pigs, together with Freund's complete adjuvant (Gibco-Biocult Ltd, Paisley, Scotland), 200 μg of this preparation resulted in paralysis of the hind legs within 15 to 17 days, and death after 17 to 21 days; quantities smaller than 200 μg were uncertain in their action. A highly purified preparation of myelin basic protein (MBP) was also isolated from human brain according to a method devised by Dr J. P. Dickinson (personal communication). This was prepared as follows: myelin was isolated from the white matter of human brain by sucrose-gradient centrifugation, and defatted with acetone, according to the method of Eylar *et al.* (1969). The defatted myelin was extracted for 12 h with dilute HCl, pH 1·5–2·0, and the solid residue removed by centrifugation and discarded. The supernatant was passed through DEAE cellulose (Whatman) at neutral pH, the MBP remaining in the filtrate under these conditions. The filtrate was adjusted to 0·2 M in Na acetate and passed through a column (2·5 × 30 cm) of CM cellulose (Whatman) equilibrated with 0·2 M Na acetate. A Na acetate gradient of 0·2 to 1·0 M was applied to the column, the MBP being eluted at an Na acetate concentration of 0·5 to 0·7 M. Injection of 5 μg of this preparation into guinea-pigs resulted in paralysis of the hind legs, incontinence and death within 14 to 18 days. Protein concentrations were determined by the biuret method of Gornall *et al.* (1949) *vs* a serum-albumin standard.

*Indicator cells.*—Sheep erythrocytes, tanned and stabilized with sulphosalicylic acid, were obtained as a lyophilized preparation from Behring-Werke A.G., Marburg-Lähn, West Germany. An aliquot was freshly prepared for use each day by suspension in HBSS, washing $\times 3$ with HBSS, and adjusting to a final concentration of $100 \times 10^6$ cells/ml.

*Preparation of solutions.*—All solutions were prepared with double-glass-distilled water and "Analar" reagents (BDH Ltd, Liverpool). HBSS and BEM were prepared in the laboratory without phenol red, and were adjusted to a pH of 7.30 with sterile 4% bicarbonate solution immediately before use. BEM was prepared with amino acid and vitamin supplements (Flow Laboratories, Irvine, Scotland). The conductivity of the BEM was routinely checked and determined to be $1{\cdot}596 \pm 0{\cdot}014 \times 10^{-2}$ mho/cm at 25°C (Model MCl Mk V, Electronic Instruments Ltd, Chertsey).

*Electrophoretic measurements.* — Except where otherwise stated, $5 \times 10^6$ lymphocytes were incubated, in a screw-top 8 ml tube, with 1 mg of BE in a final volume of 3 ml, and at a temperature of $27° \pm 0{\cdot}05$°C. The period of this pre-incubation is noted in the appropriate Figures. On termination of pre-incubation the tubes were centrifuged (300 *g* max. 10 min) and the supernatants transferred to a fresh tube containing $25 \times 10^6$ tanned and stabilized sheep erythrocytes in 0·25 ml of HBSS, and incubated at 27°C for 60 min before electrophoretic mobility measurements.

The electrophoretic mobility of the erythrocytes was determined in a cytopherometer (Zeiss, Oberkochen/Wuertt, West Germany) with modified electrode chambers and Ag/AgCl/KCl electrodes (Cam-Apparatus, Impington, Cambridge). The solution in the electrode chambers was 0·15 M KCl to avoid the problems associated with a more concentrated solution diffusing into the sample chamber. The stability of the instrument was much enhanced by the substitution of the modified chambers for those originally fitted, and previous problems with vibration were virtually eliminated.

All measurements were carried out at a potential difference of 155 V and current of 9 mA, and a temperature of $27° \pm 0{\cdot}05$°C. The time for each of 15 to 20 cells from each sample to cross one square of the microscope grid was determined, each cell being timed in both directions of the field (1 grid square= 23·54 μm). Any measurements in which the difference in timings for the two directions of the field was greater than 15% were discarded. The image of the front stationary plane of the cell, together with that of the microscope grid, was displayed on a T.V. monitor for mobility measurements; cells in focus in the stationary layer were chosen at random for measurement. The set of paired timings for each sample were averaged, and a percentage calculated as:

$$100\left(\frac{T_2 - T_1}{T_1}\right) = \%\ \text{slowing}$$

where $T_1$ is the average time for a sample of erythrocytes without addition, and $T_2$ is the average time for a sample of erythrocytes with addition of BE, with or without pre-

FIG. 1.—Effect of BE concentration on the electrophoretic mobility of tanned erythrocytes (EME) after pre-incubation of BE with or without lymphocytes. (a) 4h pre-incubation. (b) 20h pre-incubation. (▲—▲) pre-incubated without lymphocytes. (●—●) pre-incubated with lymphocytes from patients with malignant disease. (○—○) pre-incubated with lymphocytes from normal controls. Other conditions as stated in text. Each point shown is the average of triplicate determinations using 3 different lymphocyte donors. Curves fitted visually to experimental points.

FIG. 2.—Decrease in capacity of BE to reduce EME when pre-incubated with lymphocytes; effect of lymphocyte number. Lymphocytes from patients with malignant disease: (●—●) 4h pre-incubation. (○—○) 20h pre-incubation. Lymphocytes from normal controls: (▲—▲) 4h pre-incubation. (△—△) 20h pre-incubation. Other conditions as stated in text. Each point shown average of duplicate determinations using different lymphocyte donors. Curves fitted visually to experimental points.

FIG. 3.—Decrease in capacity of BE to reduce EME when pre-incubated with lymphocytes; effect of time of pre-incubation. (▲—▲) pre-incubated without lymphocytes. (●—●) pre-incubated with lymphocytes from patients with malignant disease. (○—○) pre-incubated with lymphocytes from normal controls. Each point represents a determination on one subject. Other conditions as stated in text. Top curve fitted by regression analysis. Two bottom curves: fitted visually to experimental points up to 8 h; above 8 h but excluding 48 h, fitted by regression analysis.

incubation with lymphocytes. In order to avoid, as far as possible, operator bias of the results, the following coding procedure was adopted: in the case of the data of Figs. 1 and 2 the operator was aware of the source of the lymphocytes used in a particular experiment, but the tubes were coded so that the treatment protocol of the individual samples was not known to the operator when the measurements were carried out. For the data presented in Fig. 3 and the Table, several blood samples were taken from cancer patients and normal controls during a morning, and were coded before being passed to the laboratory. Thus the operator was not aware of the category to which the lymphocyte donor belonged when the measurements were carried out.

### RESULTS

### *Effect of BE on erythrocyte mobility*

The electrophoretic mobility of fresh sheep erythrocytes is 1·14 μm/sec/V.cm (Seaman & Uhlenbruck, 1963); this compares with the value determined for the tanned and stabilized cells in the preparation used in this study of 1·20$\pm$0·03 μm/sec/V.cm.

In order to provide a baseline for subsequent measurements of the effect of pre-incubation of BE with lymphocytes, the effect of BE alone on the electrophoretic mobility of the erythrocytes (EME) used was determined. When the EME was measured after incubation for 1 h at 27°C with different concentrations of BE, results essentially identical to those shown in Figs. 1A and 1B (top lines) were obtained. Thus, in contrast to macrophages, tanned and stabilized erythrocytes are slowed even by low levels (30 μg/ml) of BE.

### *Effect of pre-incubation of BE with lymphocytes*

To determine the effect of pre-incubation of the BE with lymphocytes, a series of samples was prepared containing varying amounts of BE together with lymphocytes from either normal subjects or cancer patients; controls contained BE in medium

TABLE.—*Residual percentage slowing of tanned erythrocytes for the series of* 51 *patients with malignant disease tested, showing results related to site of disease, and also average for the* 22 *normal subjects tested*

| Diagnosis* | Number tested | Percentage slowing† |
|---|---|---|
| *Patients with malignant disease:* | | |
| Breast | 1 | 68·67 |
| Bladder | 1 | 58·69 |
| Parotid (right) | 1 | 47·82 |
| Prostate (skin involvement) | 1 | 39·07 |
| Anus (anal verge) | 1 | 36·23 |
| Breast | 12 | 36·05±7·39 |
| Cervix | 9 | 38·36±5·09 |
| Larynx | 1 | 38·22 |
| Bladder | 1 | 37·63 |
| Bronchus | 7 | 36·74±3·77 |
| Tongue | 1 | 31·71 |
| Prostate | 1 | 30·00 |
| Rectum | 1 | 28·86 |
| Colon | 1 | 27·13 |
| Bladder | 1 | 23·87 |
| Ovary | 1 | 21·71 |
| Bronchus (bony mets.) | 1 | 19·88 |
| Rectum | 1 | 19·53 |
| Bladder | 1 | 17·08 |
| Penis‡ | 1 | 16·63 |
| Rectum+bronchus | 1 | 15·53 |
| Prostate | 1 | 13·97 |
| Teratoma‡ | 1 | 13·64 |
| Urethra | 1 | 9·34 |
| Seminoma | 1 | 5·71 |
| Breast | 1 | 5·45 |
| | 51 | |
| *Normal controls* | | |
| 14 male, 8 female ages 22 to 50 | 22 | 9·65±3·03 |

* Subjects for testing selected at random from patients with known malignant disease attending prescription clinics at Cookridge Hospital before radiotherapy.

† 16–24 h pre-incubation of BE with $5\times10^6$ lymphocytes; other conditions as in text.

‡ After surgery, possibly no tumour present.

only. After pre-incubation for 4–20 h the samples were centrifuged, and supernatants and controls transferred to tubes containing an aliquot of erythrocytes, incubated at 27°C for 1 h, and the EME determined. In all cases it was found that the supernatants from those tubes containing lymphocytes caused a lesser reduction of the EME than did the controls, whose slowing capacity was essentially unchanged by pre-incubation (Figs. 1A and 1B). This loss of slowing capacity increased with time of pre-incubation, so that supernatants from samples pre-incubated for 20 h with lymphocytes from normal subjects had lost essentially all capacity to reduce EME. Moreover, for comparable periods of pre-incubation, supernatants from samples containing lymphocytes from normal subjects had lost considerably more slowing capacity than had those containing lymphocytes from cancer patients (compare centre and bottom lines, Figs. 1A and 1B). On no occasion, even with BE concentrations (30 $\mu$g/ml) and times of pre-incubation (1 h) comparable with the MEM test, was it found that supernatants from tubes containing BE plus lymphocytes from cancer patients caused EME reduction greater than that caused by an equal concentration of BE alone, pre-incubated without lymphocytes. On the contrary, the reduction in the EME could be attributed in all cases to the BE alone, and the effect of pre-incubation of the BE with lymphocytes was to cause a reduction in the slowing capacity of the BE. These experiments thus suggest that either the erythrocytes used were inherently incapable of responding to slowing factor released by sensitized lymphocytes on stimulation by the BE, or, alternatively, that the erythrocytes were saturated by the BE and therefore rendered incapable of responding. Supernatants from lymphocytes incubated without BE were also tested, but were found to have essentially no effect on the EME, no increase or decrease being observed, irrespective of the lymphocyte source.

The experiments described above demonstrated, however, a differential effect which might be used to identify subjects with malignancy, insofar as all measurements pointed to the fact that the loss of slowing capacity by the BE was greater when the lymphocytes were from normal subjects than when the lymphocytes were from cancer patients. As further results were accumulated it became apparent that the slowing capacity (residual slowing capacity) of the BE which remained after pre-incubation with lymphocytes from either cancer patients or normal subjects was dependent on the BE concentration, lymphocyte number, temperature, and time of pre-incubation. In order, therefore, to be able to employ optimal conditions for the greatest difference between results for normal subjects and for cancer patients, the influence of these parameters would have to be investigated, and the following sections describe the experimental conditions that were determined for use in the test.

*Effect of purified MBP*

The effect of substituting a highly purified preparation of MBP for the BE was also determined. At equal concentrations, the MBP was found to possess ~30% of the inhibitory capacity of the BE; however, in contrast to the crude BE, this inhibitory capacity was found to be stable and was not lost in pre-incubation with lymphocytes. In agreement with the results obtained for the BE, however, no reduction in the EME was observed which was not attributable to the MBP alone. At concentrations comparable to those used in the MEM test (30 μg/ml) the MBP had little if any effect on the EME, which suggests that, had the erythrocytes used been capable of responding to slowing factor, they would have done so under these conditions.

*Effect of BE concentration*

The relationship between the BE concentration and the inhibition of EME is shown in detail in Figs. 1A and 1B. The top lines present results for controls pre-incubated for 4 h (Fig. 1A) and 20 h (Fig. 1B) without the addition of lymphocytes. The centre lines present the results obtained when the pre-incubation was carried out with the addition of lymphocytes from patients with malignant disease, while the bottom lines present results obtained when the pre-incubation was with lymphocytes from normal subjects. The results shown suggested that a BE concentration of 300–400 μg/ml would result in optimal differentiation between the two groups, and 333 μg/ml was chosen as representing a 100 μl aliquot of a 10 mg/ml solution in a final volume of 3 ml.

It will be noted that as the BE concentration is increased above 400 μg/ml the effect of pre-incubation with lymphocytes is gradually decreased, and is essentially lost at a concentration of 1 mg/ml of BE.

*Effect of number of lymphocytes*

As lymphocytes from patients with malignant disease were less effective than those from normal controls in decreasing the level of slowing caused by a given concentration of BE, it was determined to what degree the number of lymphocytes in the pre-incubation mixture influenced the extent of the reduction. The results obtained are shown in Fig. 2 for a 4 h and a 20 h pre-incubation. When BE was pre-incubated with increasing numbers of lymphocytes from normal controls it was found that the slowing capacity of the BE reached its minimum, with about 9% slowing remaining, when pre-incubated with $4–5 \times 10^6$ lymphocytes, and remained nearly constant thereafter. In contrast, when the pre-incubation was with lymphocytes from patients with malignant disease, the decreased level of slowing due to pre-incubation with $15 \times 10^6$ lymphocytes was still above the value of 9% achieved with lymphocyte numbers of $5 \times 10^6$ and above from normal subjects. However, it is clear from Fig. 2 that addition of a sufficiently large number of lymphocytes from cancer patients will achieve the same decrease in slowing capacity of the BE as that reached by lymphocytes from normal controls.

Fig. 2 shows that the maximum difference between the two groups is close to the point where pre-incubation of the BE is with $5 \times 10^6$ lymphocytes, and this value was therefore chosen for routine measurements. This choice was also influenced by the fact that the number of lymphocytes isolated from 10 ml blood samples from patients with malignant disease is usually between 5 and $10 \times 10^6$.

*Effect of pH*

Within the pH range 5·5–8·5 no change was observed in the inhibition of the mobility of sheep erythrocytes by BE, or the effectiveness of lymphocytes of either group in reducing the level of this inhibition on pre-incubation with the BE.

*Effect of time of pre-incubation*

As shown in Figs. 1 and 2, increased time of pre-incubation of the BE with lymphocytes resulted in increased loss of its capacity to reduce the EME. A more detailed study of the time-dependency of the effect of pre-incubation of the BE with lymphocytes is presented in Fig. 3. This shows that the pre-incubation of BE for up to 26 h without addition of lymphocytes, but under otherwise identical conditions, resulted in an average decrease of 3% in the slowing capacity of the BE. With addition of lymphocytes from patients with malignant disease, and a 16–24 h pre-incubation, the slowing capacity of the BE was decreased from 73% to ~35%. When lymphocytes from normal controls were pre-incubated with the BE for 16–24 h, the slowing capacity of the BE decreased to ~10%, this representing about a 25% greater decrease in slowing capacity than that observed with pre-incubation with lymphocytes from cancer patients.

A number of pre-incubations were also carried out for 48 h, but no advantage was found in exceeding 24 h (see Fig. 3). In the case of lymphocytes from normal subjects, further decrease in the slowing capacity of the BE was of the order of a few *per cent* when the pre-incubation was prolonged from 24 to 48 h. With lymphocytes from cancer patients, a 48 h pre-incubation resulted in a further decrease of ~20% in the slowing ability of the BE, compared to the 24 h value. As this reduces the difference in residual slowing capacity between normal subjects and cancer patients, it is obviously disadvantageous.

Although differences in residual slowing between normal subjects and cancer patients were generally detectable within 4–6 h, pre-incubations of 16–24 h were found to be most convenient, and to give the most clear-cut differences between the two groups.

*Effect of temperature*

Temperatures other than 27°C for pre-incubation were also tested. After pre-incubation at 37°C for 6 h, the residual slowing capacity of the BE was found to be 20%, compared to a value of 50% for a control aliquot of the same lymphocytes pre-incubated at 27°C, but otherwise treated identically. Pre-incubation at 10°C for 24 h resulted in a residual slowing of 57%, compared to a value of 12% for a control aliquot pre-incubated at 27°C for 24 h.

It is clearly possible to carry out pre-incubations for a shorter period at 37°C if this is preferred, without appreciably affecting the difference between the two groups.

*Summary of results for subjects tested*

The results obtained for the group of patients with malignant disease and for the normal controls are summarized in the Table, which shows the residual slowing capacity of the BE associated with a particular disease site. These values were obtained after 16–24 h pre-incubation of the BE with lymphocytes under standard conditions. If values greater than 15% are arbitrarily taken as positive for malignant disease, and values less than 15% as negative, then 46/51 gave a correct positive reaction, although 3 were only marginally within this group, 5/51 gave a false negative reaction, 22/22 gave a correct negative reaction, with no false positives.

### DISCUSSION

Reference to the data presented here shows that our results, although differing in detail, are essentially consistent with those of Porzsolt *et al.* (1975), and other groups (Douwes *et al.*, 1976; Nitzschke *et*

*al.*, 1977) who have used tanned and stabilized erythrocytes, and thus support their conclusions on the use of these cells in a test which can apparently detect the presence of malignancy. Although our results are consistent with theirs we differ from these authors in our interpretation of the phenomenon on which they are based. They evidently believe that this is a modification of the MEM test in which tanned erythrocytes have been substituted for macrophages. The results we describe here, however, do not appear to be consistent with the involvement of a lymphokine, or, indeed, with the method depending on an immunological reaction, and we consider, therefore, that this method is basically different from the MEM test.

We have been consistently unable to detect the presence of a slowing factor with the erythrocyte preparations used, although with the pure MBP, which was known to be active in the MEM test, and which produced little or no slowing of the erythrocytes at low concentrations (30 $\mu$g/ml), this should have been possible had the erythrocytes been capable of responding. The reduction of the electrophoretic mobility of the erythrocytes (EME) appears, therefore, to be attributable only to the BE, and the effect of the incubation with lymphocytes is to *decrease* the slowing capacity of the BE. This is essentially the reverse of the situation in the MEM test, where much lower concentrations of BE are used, which do not affect the mobility of the macrophages, and where slowing is attributed to release of slowing factor by the lymphocytes on stimulation by BE. Furthermore, the pure MBP, although at least 20-fold more potent in inducing experimental allergic encephalomyelitis in guinea-pigs than the BE, was much less effective in slowing erythrocytes, and this effect was not lost on pre-incubation with lymphocytes. This supports the view that the function of the BE is, in this case, not immunological, and it also suggests that the method depends on some constituent of the BE other than MBP.

Results have been recently published by Harlos & Weiss (1978), however, which suggest that it may be possible to prepare erythrocytes which respond to slowing factor. A direct comparison with our results and with those of Porzsolt *et al.* (1975) is however difficult, owing to differing experimental protocols. Their results (Harlos & Weiss, 1978) suggest that erythrocytes prepared according to their method are considerably less sensitive to slowing by BE than the preparation used by us, which may allow response to slowing factor. A final decision on this point must await the use in our system of erythrocytes prepared according to their protocol, although it does appear from the different time-scales involved that we are measuring 2 basically different phenomena.

Examination of Figs. 1–3 shows that the decrease in the capacity of the BE to reduce EME is dependent on the number of lymphocytes in the pre-incubation mixture, on the time of pre-incubation, on temperature, and on the BE concentration. All these factors are consistent with the action of the lymphocytes being to remove or destroy some constituent of the crude BE preparation, this constituent being responsible for reducing EME of the erythrocytes. Absence of this constituent from the chromatographically pure MBP preparation would explain the latter's relative ineffectiveness in slowing erythrocytes, as well as the observation that such slowing power as it has was quite stable to pre-incubation with lymphocytes. The method, therefore, depends on the observation that lymphocytes from normal controls are more effective in removing or destroying this constituent than are lymphocytes from patients with malignant disease. Thus, with appropriate selection of BE concentration, lymphocyte number, and time of pre-incubation, patients with malignant disease may be identified by the greater residual slowing action of the BE after pre-incubation with their lymphocytes, when compared with values for normal controls. The method also obviously requires the use of a relatively crude BE, at least until the possibility of isolating the appropriate constituent has been explored.

A total of 51 patients with malignant disease has been tested so far, and the range of sites of disease within this group is relatively broad, so that conclusions regarding effect of disease site, type of malignancy and tumour load on the residual slowing of the BE must necessarily be limited. In the group of 43 patients that gave positive values for residual slowing there appears to be no correlation between the values for residual slowing and tumour load, as light to heavy tumour loads are distributed randomly throughout the range of slowing values. Of the 8 patients who gave residual slowing values in, or only marginally above, the range for normal controls, 2 had undergone surgery and possibly had no tumour present, the remaining 6 had light to moderate tumour loads, and thus do not differ in this respect from the subjects who gave a positive result.

A double-blind study involving a larger number of subjects is at present being conducted, in order to assess the usefulness of the method as a means of determining the presence of malignant disease, and this will also provide an opportunity for studying the effect of the disease parameters on the values for residual slowing. The present series, in which 43/51 subjects with malignant disease gave an unequivocal positive reaction, does, however, represent a highly significant separation of results, with a high probability of correctly assigning a subject to the correct "malignant" or "normal" group, with few false-positive errors.

No technical difficulties have so far been encountered with the test, and it does not appear to be sensitive to the experimental conditions employed, provided normal care is taken in quantitation. The loss of slowing capacity of the BE is a continuous process in the presence of lymphocytes, and is thus time- and temperature-dependent to a greater extent than in the case of the MEM test, and these factors must therefore be carefully controlled. The cytopherometer operator is not faced with the task of choosing a particular type of macrophage for measurement, as in the MEM test, but can choose erythrocytes at random from those in focus in the stationary layer. Thus operator judgement in choosing the correct type of macrophage for measurement is not a factor in this method. The problems of maintaining a guinea-pig colony and obtaining peritoneal exudates containing appropriately responsive macrophages are also avoided.

This work was carried out with the sole financial support of the Yorkshire Cancer Research Campaign, and was directed by Professor C. A. F. Joslin. We are indebted to Dr Sheila Corbett for blood samples from her patients and to the Consultant Staff of Cookridge Hospital for permission to approach their patients for blood samples; also to Dr J. P. Dickinson for help and advice, and for making available his method for isolating myelin basic protein, and to Mr Denzil Evans of Hoechst Pharmaceutical Division for obtaining and supplying us with tanned and stabilized sheep erythrocytes from Behring Werke A.G., Marburg-Lähn, West Germany.

### REFERENCES

Arvilommi, H., Dale, M. M., Desai, H. N., Mongar, J. L. & Richardson, M. (1977) Failure to obtain positive MEM tests in either cell-mediated immune conditions in the guinea-pig or in human cancer. *Br. J. Cancer*, **36**, 545.

Bagshawe, K. D. (1977) Workshop on macrophage electrophoretic mobility (MEM) and structuredness of cytoplasmic matrix (SDM) tests. *Br. J. Cancer*, **35**, 701.

Caspary, E. A. & Field, E. J. (1965) An encephalitogenic protein of human origin; some chemical and biological properties. *Ann. N.Y. Acad. Sci.*, **122**, 182.

Caspary, E. A. & Field, E. J. (1971) Specific lymphocyte sensitization in cancer: is there a common antigen in human malignant neoplasia? *Br. Med. J.*, **ii**, 613.

Crozier, E. H., Hollinger, M. E., Woodend, B. E. & Robertson, J. H. (1976) An Assessment of the Macrophage Electrophoretic Mobility Test (MEM) in Cancer Diagnosis. *J. Clin. Path.*, **29**, 608.

Douwes, F. R., Hanke, R. & Mross, K. (1976) Der Elektrophorese-Mobilitäts-Test (EMT) in der Diagnostik von Malignomen. 82 *Verhandlung der deutschen Gesellschaft für innere Medizin, Weisbaden.*

Eylar, E. H., Salk, J., Beveridge, G. C. & Brown L. V. (1969) Experimental allergic encephalomyelitis: an encephalitogenic basic protein from bovine myelin. *Arch. Biochem. Biophys.*, **132**, 34.

Field, E. J. & Caspary, E. A. (1970) Lymphocyte sensitization: an *in vitro* test for cancer. *Lancet*, **ii**, 1337.

Forrester, J. A., Dando, P. M., Smith, W. J. & Turberville, C. (1977) Failure to Confirm the

Macrophage Electrophoretic Mobility Test in Cancer. *Br. J. Cancer*, **36**, 537.

GOLDSTONE, A. H., KERR, L. & IRVINE, W. J. (1973) The Macrophage Electrophoretic Migration Test in Cancer. *Clin. Exp. Immunol.*, **14**, 469.

GORNALL, A. G., BARDAWILL, C. J. & DAVID, M. M. (1949) Determination of Serum Proteins by Means of the Biuret Reaction. *J. Biol. Chem.*, **177**, 751.

HARLOS, J. P. & WEISS, L. (1978) Comparison between the macrophage electrophoretic mobility (MEM) and the fixed tanned erythrocyte electrophoretic mobility (FTEEM) tests in the detection of cancer. *Int. J. Cancer*, **21**, 413.

LEWKONIA, R. M., KERR, E. J. L. & IRVINE, W. J. (1974) Clinical evaluation of the macrophage electrophoretic mobility test for cancer. *Br. J. Cancer*, **30**, 532.

MOORE, M. & LAJTHA, L. G. (1977) Lymphocyte responses to human tumor antigens: their role in cancer diagnosis. In *International Review of Experimental Pathology*, Ed. G. W. Richter & H. A. Epstein. London: Academic Press, p. 17.

NITZSCHKE, U., ZWERGEL, T. & LAMPERT, F. (1977) Electrophoretic mobility (EM)-test for childhood cancer diagnosis. *Eur. J. Pediat.*, **126**, 163.

PORZSOLT, F., TAUTZ, C. & AX, W. (1975) Electrophoretic mobility test: I. Modifications to simplify the detection of malignant diseases in man. *Behring Inst. Mitt.*, **57**, 128.

PREECE, A. W. & LIGHT, P. A. (1974) The macrophage electrophoretic mobility (MEM) test for malignant disease. Further clinical investigations and studies on macrophage slowing factors. *Clin. Exp. Immunol.*, **18**, 543.

PRITCHARD, J. A. V., MOORE, J. L., SUTHERLAND, W. H. & JOSLIN, C. A. F. (1972) The macrophage electrophoretic mobility (MEM) test for malignant disease: an independent confirmation. *Lancet*, **ii**, 627.

PRITCHARD, J. A. V., MOORE, J. L., SUTHERLAND, W. H., & JOSLIN, C. A. F. (1973) Evaluation and development of the macrophage electrophoretic mobility (MEM) test for malignant disease. *Br. J. Cancer*, **27**, 1.

PRITCHARD, J. A. V., MOORE, J. L., SUTHERLAND, W. H. & JOSLIN, C. A. F. (1976) Clinical assessment of the MOD–MEM cancer test in controls with non-malignant disease. *Br. J. Cancer*, **34**, 1.

RAWLINS, G. A., WOOD, J. M. F. & BAGSHAWE, K. D. (1976) Macrophage electrophoretic mobility (MEM) with myelin basic protein. *Br. J. Cancer*, **34**, 613.

SEAMAN, G. V. F. & UHLENBRUCK, G. (1963) The surface structure of erythrocytes from some animal sources. *Arch. Biochem. Biophys.*, **100**, 493.